# Prevalence and determinants of contraception use in Pakistan: trend analysis from the Pakistan Demographic and Health Surveys (PDHS) dataset from 1990 to 2018

**DOI:** 10.12688/f1000research.55204.1

**Published:** 2021-08-11

**Authors:** Salima Meherali, Anna Ali, Asif Khaliq, Zohra S. Lassi

**Affiliations:** 1 Faculty of Nursing, University of Alberta, Edmonton, Canada, Alberta, T6G 1C9, Canada; 2 Robinson Research Institute, The University of Adelaide, Adelaide, Australia; 3 School of Public Health and Social Work, Queensland University of Technology, Brisbane, Queensland, Austria

**Keywords:** Pakistan, contraception, demographic and health survey

## Abstract

**Background:** In developing countries, pregnancy and childbirth are the leading causes of death among women. In this context, family planning and access to contraceptives are crucial for reducing pregnancy-related morbidity and mortality. Therefore, we aimed to look into the trends of contraception and determinants of contraceptive use in Pakistan.

**Methods:** This study used data for women of reproductive age from four Pakistan Demographic and Health Surveys datasets. Contraception was the outcome variable, whereas, women’s and partner’s education, occupation, wealth quintile, region, place of residence, and exposure to family planning messages were the explanatory variables. Pooled prevalence was estimated using SUMARI and regression analysis was undertaken using SPSS to produce an adjusted prevalence ratio with 95% confidence intervals.

**Results:** Data of 40,259 ever-married women of reproductive age (EMWRA) was analysed. Of the total EMWRA, 30% were using contraception. Of these, 26% were using traditional methods and 74% were using modern methods. The most common method of contraception was condoms (30.5%). The pooled prevalence of contraception used was 29.5% (95% CI 29.1 to 30.0). Through multivariate analysis, women's age, place of residence, region, wealth index, women’s education, their working status, and exposure to family planning messages were found to be significant determinants of contraception usage.

**Conclusions:** There is a noticeable gap regarding awareness and uptake of contraception leading to low contraceptive use among women in Pakistan. In the light of our results, it is important to highlight the importance of girl’s education for building awareness and empowerment.

## Introduction

Pregnancy and childbirth are the leading causes of death among girls and women aged 15 to 49 years in many low and middle-income countries (LMICs) ( World Health Organization [WHO], 2017). In this context, family planning and access to contraceptives are crucial for reducing pregnancy-related morbidity and mortality, improving the health outcomes of young girls and women and their children, and reducing the related social and economic costs of early pregnancy ( Chandra-Mouli *et al*., 2017). Although significant progress has been made in improving coverage of family planning services worldwide ( Cahill *et al*., 2018; MacQuarrie, 2014) there is still a large gap in relation to effectively meeting the contraceptive needs and family planning goals in LMICs ( Chandra-Mouli *et al*., 2017; MacQuarrie, 2014; Dennis *et al*., 2017; Ewerling *et al*., 2018).

Pakistan is an LMIC situated in the South Asian region and shares the highest population growth rate i.e. 2% per year in South Asia (The World Bank, 2019). Progress towards accomplishing the United Nations’ Sustainable Development Goals (SDG) to increase the contraceptive prevalence rate (CPR) to 55% by 2015 remained unachievable for Pakistan. One of the pivotal reasons for the high population growth rate of Pakistan is the unmet need for family planning ( Nyoni, 2018). The government of Pakistan and private health sectors have been continuously struggling to bring down population growth by improving the availability of family planning services. Although progress has been made to reduce the fertility rate from seven children per woman in 1970 ( Shah *et al*., 1986) to 3.6 children per woman in 2020, the acceptability and use of contraceptives in the country are low (A. Mahmood & Sultan, 2006). According to the World Bank report (2015), the average CPR in South Asian countries is 53%, and Pakistan has the lowest rate of 35%. Many potential barriers exist to contraceptive use among women of reproductive age (WRA) in Pakistan such as the social, cultural, and perceived religious unacceptability of contraception, lack of knowledge and awareness of contraception, cost of contraceptives, and access to contraceptive services ( Asif and Pervaiz, 2019; Memon & Jonker, 2018; N.Z. Shah *et al*., 2020; Dasgupta *et al*., 2019).

Low CPR increases the risk of unplanned pregnancies, teenage pregnancies, abortions, and thereby resulting in poor maternal and child health outcomes ( Wulifan *et al*., 2017). Moreover, the low CPR also produces a drastic effect on the economy of a nation. Currently, Pakistan is facing issues related to inflation, poverty, unemployment, and other related economic crises ( Nyoni, 2018). Under such conditions, it is essential to know about the trends and determinants of CPR in Pakistan from the nationally available datasets. Knowing the trends and determinants of contraceptive prevalence among WRA will aid in understanding and planning appropriate interventions and policies for the promotion of contraceptive use. This in turn helps the nation to control the population outgrowth and other economic issues.

The objectives of this study were:

• To determine the prevalence of contraception among WRA in Pakistan
• To identify the factors leading to low CPR among WRA in Pakistan

## Methods

Datasets from the Pakistan Demographic and Health Surveys (PDHS) were used in this study (NIPS, 2019). These surveys include information on the health, demographic and socioeconomic characteristics of the representative sample of Pakistan. From 1990 until 2018 Pakistan has implemented four Demographic and Health Surveys (DHS) under the AEGIS of the National Institute of Population Studies (NIPS) and Pakistan Bureau of Statistics (PBS) ( National Institute of Population Studies, 1992). For the current study, we have used secondary data of all the DHS conducted in Pakistan regarding the use of contraception by ever-married women of reproductive age (EMWRA) aged between 15 to 49 years.

A multistage stratified cluster systematic sampling technique was used in these surveys. After stratification of all provinces on the urban and rural population, Enumeration Blocks (EB) (the cluster of 200 to 250 households (HH)) were selected followed by random selection of 20-30 HH from each EB. The total number of EMWRA was 40,259; 6,611 in 1990-91 PDHS; 9,177 in 2006-07 PDHS; 11,763 in 2012-13 PDHS; and 12,708 in 2017-18 PDHS.

The outcome variable of this study was the use of contraception both modern (such as a pill, intrauterine device, injection, condom, female sterilization, implant) and traditional (such as withdrawal, and lactation) methods. Use of contraception was defined by using the information of current methods of contraception used by study participants at the time of the interview. Women using any modern or traditional methods of contraception were grouped. The information regarding the use of contraception was collected verbally by the interviewers in the PDHS. Information on types and methods of contraception were also collected verbally and this information is included in the analyses. Data on the decision of using contraception were available for two years only 2012-13 and 2017-18 and data regarding the decision of not using contraception were available for the year 2017-18 only. Respondent’s age was available in seven categories which were merged into four categories as 15-24, 25-34, 35-44, and 45 and above. Respondent’s and partner’s education were coded into four categories: no education, primary, secondary, and higher. Both respondent’s and their partner’s occupations were coded as: not working, professional, services/sales, agricultural, skilled, and unskilled. To avoid a small cell count, occupation categories for respondents were merged into working and not working; while for their partner was merged into: not working, unskilled, skilled, and professional categories for the partner. The place of residence was coded as urban or rural; the region was coded into Punjab, Sindh, Khyber Pakhtunkhwa (KPK), Baluchistan, and Islamabad Capital Territory (ICT). Wealth index was constructed using principal component analysis on assets-ownership including land and livestock with a range of socio-economic factors including income, type of flooring, availability of electricity, radio, television, telephone and refrigerator, type of vehicle, persons sleeping per room, household construction, utilities, source of drinking water and sanitation facilities, ownership of agricultural land, domestic servants. and categorized as five wealth quintiles: poorest, poorer, middle, richer, and richest; and exposure to family planning messages via radio, TV, and newspaper were merged and categorised as yes and no to avoid small cell counts.

All analysis was done in SPSS version 26 (Jiang & Hardee, 2014) and pooled prevalence was estimated from Joanna Briggs Institute’s SUMARI ( Babalola, 2014). Frequency and percentage of categorical variables and mean with standard deviation (SD) of continuous variables were reported. The use of contraception was defined by using the information of current methods used by study participants. Women using any method (traditional or modern) of contraception were grouped. The rate of contraception usage was determined for each year (1990-91, 2007-08, 2012-13, and 2017-18) separately, and later pooled prevalence was estimated. Prevalence ratios were estimated for socio-demographics, media exposure, and use of contraception using cox regression. All variables with borderline statistical significance (p < 0.25) were considered as potential confounding variables. The determinant of contraception usage is reported as prevalence ratio (PR) with a 95% confidence interval (CI). Multivariable regression models were used to produce covariate-adjusted PR (APR) and 95% CIs. R is an open access software that could also be used for performing this analysis.

## Results

Data of 40,259 EMWRA was analysed. In total, 29.4% were in the age group 25-29 years and 23.4% were between 30-34 years. More than half (57.4%) were from rural areas of Pakistan and around one-third were from Punjab and Sindh (29.8% and 22.8%), respectively. Around half of the women (44.7%) were from the poorest and poor wealth quantile. More than half were not educated (60.7%) and 80.8% were not working. With respect to their partner, almost one-third (33.6%) were not educated, and almost (97%) all were working in some capacity. Of all the women, a quarter heard about family planning on the TV (25.3%), and a small percentage on the radio (7.6%) and newspaper (3.7%) ( Table 1).

**Table 1.** Sociodemographic characteristics of women with respect to contraception usage from 1990-2018.

Socio-demographic characteristics	Total women N = 40259 (%)	Using contraception n = 12078 (%)	Not using contraception n = 28181 (%)	P value
**Age**				
15-19	1084(3.5)	172(1.8)	912(4.2)	<0.001
20-24	5941(19.1)	1462(15.3)	4479(20.8)	
25-29	9172(29.4)	2844(29.7)	6328(29.3)	
30-34	7294(23.4)	2647(27.6)	4647(21.5)	
35-39	4666(15.0)	1652(17.2)	3014(14.0)	
40-44	1988(6.4)	600(6.3)	1388(6.4)	
45-49	1000(3.2)	201(2.1)	799(3.7)	
**Place of residence**				
Urban	17057(42.6)	6424(53.2)	10633(38.0)	<0.001
Rural	23019(57.4)	5652(46.8)	17367(62.0)	
**Regions**				
Punjab	11941(29.8)	4249(35.2)	2692(27.5)	<0.001
Sindh	9130(22.8)	2363(19.6)	6767(24.2)	
KPK	7779(19.4)	2276(18.8)	5503(19.7)	
Baluchistan	5358(13.4)	907(7.5)	4451(15.9)	
GB	2008(5.0)	737(6.1)	1271(4.5)	
ICT	1519(3.8)	851(7.0)	668(2.4)	
AJK	1320(3.3)	445(3.7)	875(3.1)	
FATA	1021(2.5)	248(2.1)	773(2.8)	
Wealth index *				
Poorest	7801(23.2)	1323(11.8)	6478(28.9)	<0.001
Poorer	7249(21.5)	1908(17.0)	5341(23.8)	
Middle	6632(19.7)	2355(21.0)	4277(19.1)	
Richer	6079(18.1)	2646(23.6)	3433(15.3)	
Richest	5887(17.5)	2987(26.6)	2900(12.9)	
**Education**				
No education	24338(60.7)	5378(44.5)	18960(67.7)	<0.001
Primary	5288(13.2)	1921(15.9)	3367(12.0)	
Secondary	6718(16.8)	2824(23.4)	3894(13.9)	
Higher	3732(9.3)	1953(16.2)	1779(6.4)	
**Partner's education**				
No education	13389(33.6)	2909(24.2)	10480(37.8)	<0.001
Primary	6039(15.2)	1700(14.1)	4339(15.6)	
Secondary	13227(33.2)	4340(36.1)	8887(32.0)	
Higher	7136(17.9)	3081(25.6)	4055(14.6)	
**Partner's occupation**				
Not working	1197(3.0)	334(2.8)	863(3.1)	<0.001
Professional	4520(11.4)	1783(14.8)	2737(9.9)	
Skilled	24416(61.6)	7268(60.5)	17148(62.1)	
Unskilled	9506(24.0)	2628(21.9)	6878(24.9)	
**Occupation**				
Not working	32384(80.8)	9859(81.7)	22525(80.5)	0.004
Working	7677(19.2)	2209(18.3)	5468(19.5)	
**Heard family planning on Radio**				
Yes	3047(7.6)	893(7.4)	2154(7.7)	0.307
No	36992(92.4)	11168(92.6)	25824(92.3)	
**Heard family planning on Television**				
Yes	8494(25.3)	3701(33.0)	4793(21.4)	<0.001
No	25127(74.7)	7505(67.0)	17622(78.6)	
Heard family planning on Newspaper **				
Yes	895(3.1)	467(5.4)	428(2.7)	<0.001
No	23549(96.3)	8201(94.6)	15348(97.3)	
**Year of data collection**				
1990-91	6611(16.4)	859(7.1)	5752(20.4)	<0.001
2007-08	9177(22.8)	2538(21.0)	6639(23.6)	
2012-13	11763(29.2)	4287(35.5)	7476(26.5)	
2017-18	12708(31.6)	4394(36.4)	8314(29.5)	

* Data not available for the year 1990-91.

** Data not available for 1990-91 and 2007-08.

Acronyms: AJK: Azad Jammu Kashmir; FATA: Federally administered Tribal Areas; GB: Gilgit Baltistan; ICT: Islamabad Capital Territory; KPK: Khyber Pakhtunkhwa.

Of the total 12,078 women who were using contraception, more than half were of age 25-34 and were from urban areas of Pakistan (53.2%). Around one-third of the province of Punjab (35.2%) followed by Sindh (19.6%) and Baluchistan (18.8%) and half of them were from richer and richest wealth quintile (50.2%) ( Table 1).

**Figure 1.**
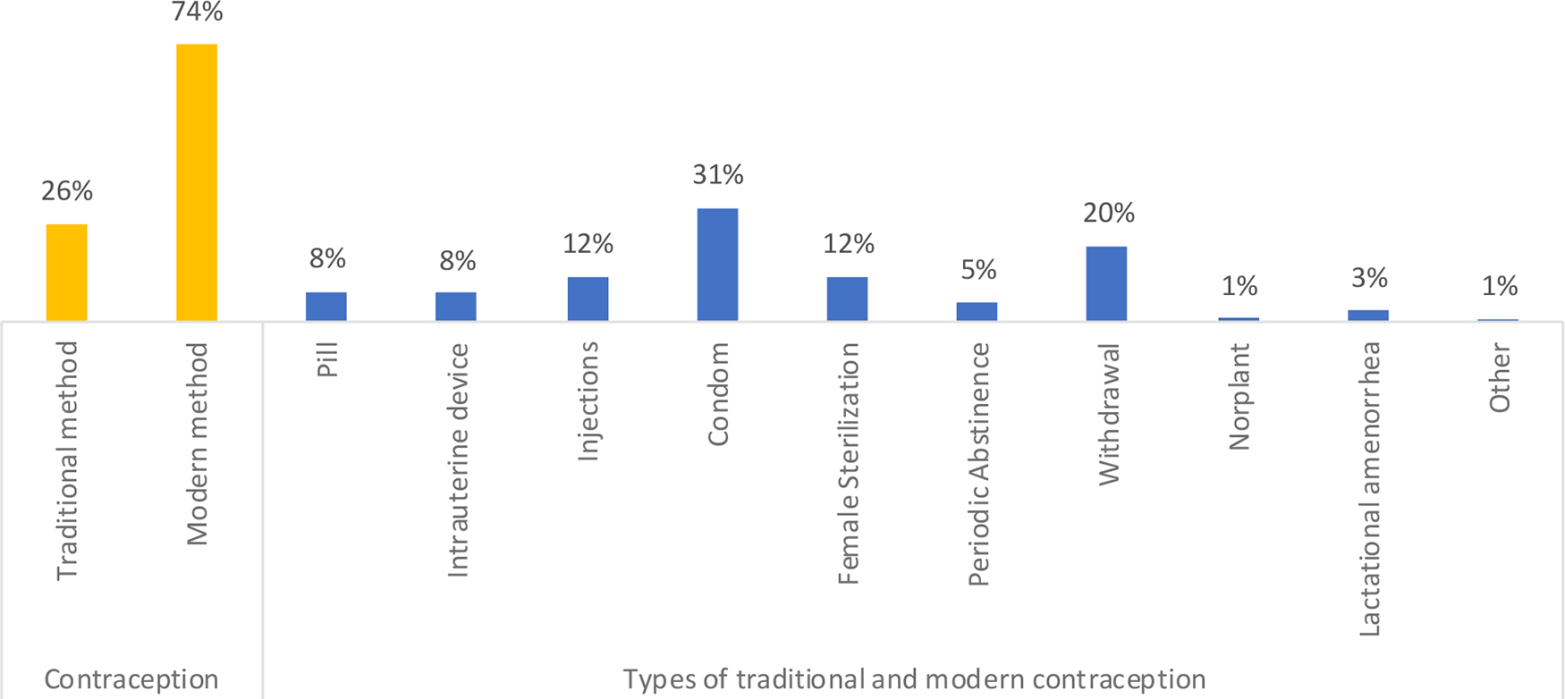
Percentage of users by type of method used at the time of the survey, 1990-2018.

**Figure 2.**
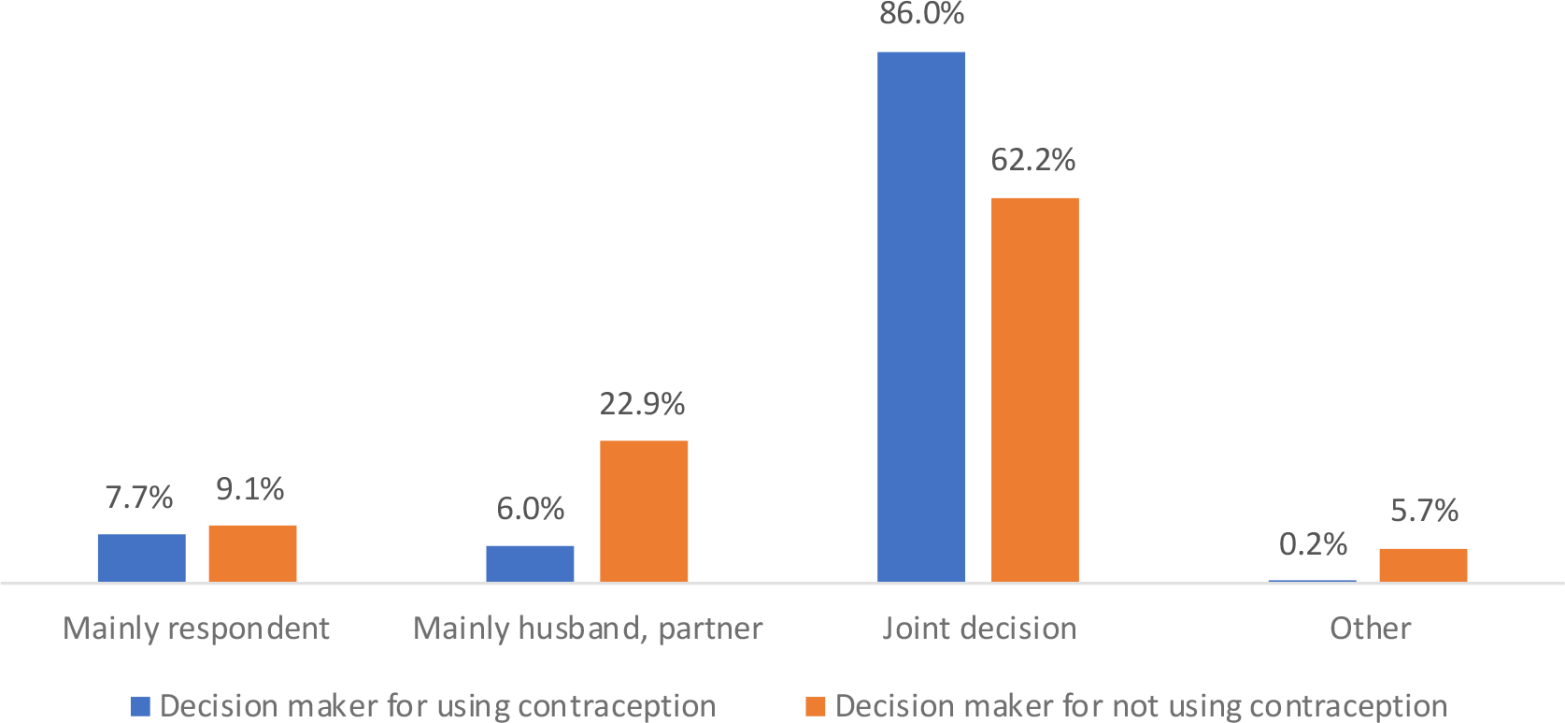
Percentage of husband or wife jointly making the decision to use or not to use contraception, 2012-2018.* *Data on the decision of using contraception were available for two years only 2012-13 and 2017-18 and data regarding the decision of not using contraception were available for the year 2017-18 only.

Of the total EMWRA, 30% were using contraception. Of these, 26% were using traditional methods and 74% were using modern methods. With respect to the individual methods, the most common method of contraception was condom (30.5%) followed by withdrawal (20.4%), female sterilization (12.0%), injections (12.1%), pills (8.0%), intrauterine device (IUD) (7.5%), periodic abstinence (5.1%), lactation amenorrhea (2.8%), Norplant (0.9%) and other methods (0.7%) ( Figure 1). With respect to the decision-making for using or not using contraception, most of them reported that it is the joint decision (86.0% vs 62.2%, respectively). However, when it is one-sided, the husband decides not to use contraception (22.9%) ( Figure 2).

**Figure 3.**
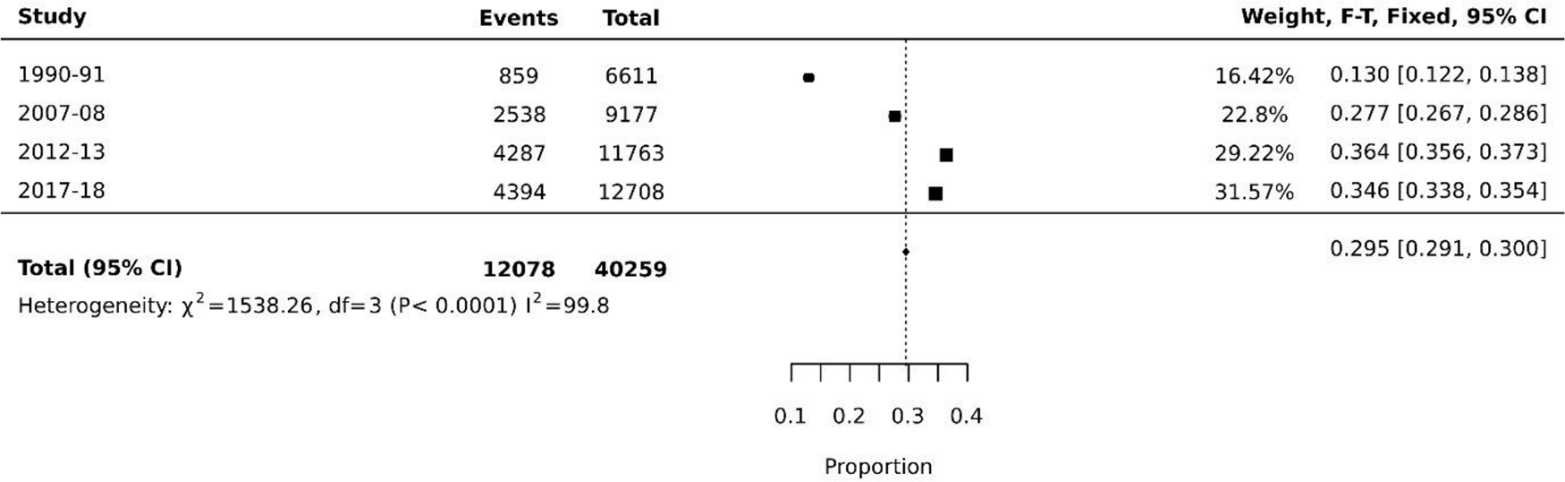
Rates of contraception usage among women of reproductive age group from 1990-2018.

The rate of contraception use was 13.0% (95% CI 12.2 to 13.8) in 1990-91 and since then the rates increased to 27.7% (95% CI 26.7 to 28.6) in 2006-07, 36.4% (95% CI 35.6 to 37.3) in 2012-13, and slightly declined to 34.6% (95% CI 33.8 to 35.5) in 2017-18. The overall pooled prevalence of contraception used was determined as 29.5% (95% CI 29.1 to 30.0) ( Figure 3).

Through multivariate analysis, it was found that women's age, place of residence, region, wealth index, education, working status, and exposure to family planning messages were significant determinants of contraception usage. The APR was significantly higher among women who were aged 45 years and above (APR 1.59; 95% CI 1.32 to 1.91) followed by women aged 35-44 years (APR 1.47; 95% CI 1.37 to 1.58) and women aged 25-34 years (APR 1.26; 95% CI 1.19-1.33) compared to women aged 15-24 years. The APR was significantly higher among women who were from urban areas (APR 1.08; 95% CI 1.02 to 1.13) compared to those who were living in rural areas. However, the APR was significantly lower among women who were from the province of Sindh (APR 0.83; 95% CI 0.72 to 0.86), KPK (APR 0.78; 95% CI 0.71 to 0.84), and Baluchistan (APR 0.58; 95% CI 0.52 to 0.65) compared to those living in ICT. The APR of contraception usage was significantly higher among women who were from the richest quantile (APR 2.10; 95% CI 1.91 to 2.32) followed by richer quantile (APR 2.02; 95% CI 1.85 to 2.21), middle quantile (APR 1.77; 95% CI 1.63 to 1.92) and poorer quantile (APR 1.43; 95% CI 1.32 to 1.55) compared to women who were from poorest quantile. The APR was higher for women who were highly educated (APR 1.08; 95% CI 1.00 to 1.16) and (APR 1.13; 95% CI 1.07 to 1.19) for those who had primary or secondary education compared to women who were not educated. With respect to women's occupation, APR was significantly higher among women who were working (APR 1.22; 95% CI 1.16 to 1.30) compared to not working women. Lastly, women who had exposure to family planning messages had significantly higher APR of contraception usage (APR 1.21; 95% CI 1.15 to 1.27) ( Table 2).

**Table 2.** Social determinants of women with respect to contraception usage.

Determinants	PR	95% CI	APR	95% CI
**Age**				
15-24	Ref			
25-34	1.36	1.29-1.44	1.26	1.19-1.33
35-44	1.60	1.50-1.70	1.47	1.37-1.58
45 and above	1.70	1.47-1.97	1.59	1.32-1.91
**Place of residence**				
Rural	Ref			
Urban	1.50	1.45-1.56	1.08	1.02-1.13
**Regions**				
Punjab	1.15	1.07-1.24	1.01	0.93-1.09
Sindh	0.81	0.78-0.87	0.83	0.72-0.86
KPK	0.68	0.63-0.73	0.78	0.71-0.84
Baluchistan	0.47	0.43-0.51	0.58	0.52-0.65
ICT	Ref			
**Wealth index**				
Poorest	Ref			
Poorer	1.50	1.40-1.61	1.43	1.32-1.55
Middle	2.08	1.95-2.23	1.77	1.63-1.92
Richer	2.58	2.42-2.76	2.02	1.85-2.21
Richest	2.98	2.80-3.18	2.10	1.91-2.32
**Education**				
No education	Ref			
Primary/secondary	1.45	1.40-1.51	1.13	1.07-1.19
Higher	1.56	1.48-1.64	1.08	1.00-1.16
**Occupation**				
Not Working	Ref			
Working	1.13	1.08-1.19	1.22	1.16-1.30
**Partner’s education**				
No education	Ref			
Primary/secondary	1.25	1.20-1.31		
Higher	1.46	1.39-1.53		
**Partner’s occupation**				
Not working	Ref			
Unskilled	1.08	0.96-1.21		
Skilled	1.28	1.14-1.43		
Professional	1.33	1.18-1.50		
**Heard family planning on social/mass media**				
No	Ref			
Yes	1.86	1.79-1.93	1.21	1.15-1.27

Acronyms: ICT: Islamabad Capital Territory; KPK: Khyber Pakhtunkhwa.

## Discussion

Family planning and planned pregnancies are crucial to the health and development of a child as well as their mothers, thereby reducing maternal and child mortality, and rates of unsafe abortions. Besides health benefits, family planning offers a range of non-health benefits that entails women empowerment, sustainable population growth, and economic development of the country. Despite major family planning initiatives by the government in Pakistan and being one of the first countries in South Asia to start a national family-planning programme ( Fikree *et al*., 2001), the total fertility rate remains high with relatively low contraception usage ( Shah *et al*., 2020; Cham *et al*., 2005).

The results of the study highlight the key contextual factors that are associated with the high prevalence of contraceptive use among EMWRA. Some of the factors that increase the likelihood of contraceptive use include the education level of mothers, their employment status, exposure to family planning messages, and overall socio-economic status. Firstly, our multivariate analysis reveals that the use of contraception was significantly higher among educated women and those who belonged to the working class.

These findings mirror the results from other studies where mainly the completion of primary and secondary school education of women was strongly correlated to lower desire for fertility (Jiang & Hardee, 2014), the greater number of antenatal visits ( Babalola, 2014), higher use of contraception and the higher probability of using family planning practices (Jiang & Hardee, 2014). Similarly, studies from India and Bangladesh show that consultation with doctors particularly about family planning was more common among working women than the unemployed ones (Jiang & Hardee, 2014; Islam *et al*., 2016). This could be attributed to the impact of education which leads to women empowerment through employment that further influences their health-seeking behaviour ( Jamali *et al*., 2018). Another explanation could be the improved decision-making ability among educated women that leads to improved insight about health problems, resulting in enhanced health-seeking behaviour ( Jamali *et al*., 2018). Furthermore, formal education exposes women to the outside world, generates awareness, and empowers them to make independent choices about family making ( United Nations Educational, Scientific and Cultural Organization [UNESCO], 2014). In contrast, illiteracy greatly reduces the modes of communication available to reach women, prohibits access to a world of ideas, and allows them access to information only through their husbands and other relatives ( León *et al*., 2014), hence influencing their freedom for using contraceptive methods.

Secondly, it was noted that the use of contraceptive or family planning methods was highly prevalent among women who heard about family planning on the TV or greatly aware of it. The most common sources of information that remained vital in promoting contraceptive usage, as highlighted in previous studies include TV, radio, printed material, and health facilities ( Qazi *et al*., 2010). Particularly, media due to its enhanced access and availability provides more opportunities for women to communicate with their friends and relatives for information regarding contraception, hence educating the community through media will increase the contraception practice rate ( Jaffery *et al*., 2019).

Thirdly, the use of contraception was reported to be higher among women living in urban areas of Pakistan. The average distance to a reproductive health facility in rural areas is larger than that to urban areas, hence access to family health services is difficult for rural women, especially without transportation or funds ( Mustafa *et al*., 2015). On the other hand, in urban areas, the proximity to health facilities and more reproductive and family planning services increases the odds of receiving more information related to family planning methods which reflects more usage of contraceptives ( Qazi *et al*., 2010).

Furthermore, the prevalence of contraception use was noted to be higher among older age women as compared to women aged between 15 to 24 years. This could be linked with the number of births per woman that influences their decisions regarding contraceptive usage. In one study conducted in Pakistan, women having three or more children were more inclined to using family planning methods compared to those who had two or fewer children ( Qazi *et al*., 2010). In addition, women’s independence, choice, and decision-making capacity increase with their increased age that may be attributed to the cultural norm whereby a newly married woman is expected to perform household chores under the supervision of her husband or mother-in-law, who is the primary decision-maker ( Hameed *et al*., 2014). Hence, such cultural factors combined with the impact of childbearing age and the high risk of mortality associated with pregnancies can potentially lead to early parenthood, unintended pregnancies among teenagers, and greater maternal and child mortality ( Patton *et al*., 2009; Nishtar *et al*., 2013).

Lastly, our findings show that using family planning methods is a conjoint decision that is strongly related to the communication between the spouses, however, disapproval for its usage mainly relies on the decision of husbands which impacts the practice of contraception among couples. Previous literature on Pakistan also underlines the role of the husband as an obstacle to family planning use by their wives ( National Institute of Population Studies, 1992; N. Mahmood & Ringheim, 1996; Agha, 2010). Such a situation might arise due to the patriarchal and patrilocal family structure in Pakistan where marriages are mostly contracted between relatives’ families and women exercise less autonomy in the extended households (N. Mahmood & Ringheim, 1996). Whereas, a man is considered the prime decision-maker and holds the financial power to implement their decisions. Therefore, generating awareness and clarifying misconceptions about family planning among men can significantly improve the use of contraception among couples. Similarly, accessibility of services and information on male methods can potentially enhance its usage, because many women, especially in rural areas, have limited mobility; and require money and permission from the husband to leave the household for traveling alone to a clinic or service outlet ( Kiani, 2003).

Despite national representation of study findings, the cross-sectional nature of the study caused biases related to the respondents. Moreover, the question asked related to the contraceptive prevalence were not timebound and due to this reason, there can be some recall biases. Moreover, some variables were available from a certain time period for example wealth index was not available for the year 1990-91. Information on hearing family planning information from the newspaper was not collected in the year 1990-91 and 2007-08. Likewise, information on the decision of using contraception was available for two years and information on the decision of not using contraception was available for one year only. The secondary nature of the data also limits the assessment of certain factors, such as the influence of family members, family structure, socio-cultural norms, and beliefs relate to the use of contraceptives. Further studies are needed which could explore the socio-cultural beliefs and the reasons for the low contraceptive prevalence rate among the women of Pakistan.

## Conclusion

The PDHS data analysis demonstrates that there is a noticeable gap regarding awareness and uptake of contraception leading to low contraceptive use among women in Pakistan. This study has identified some important determinants that significantly impact the use of contraceptives among EMWRA in Pakistan. Contraceptive use is significantly influenced by women's age, education, place of residence, region, wealth index, educational and working status of women, and exposure to family planning information on social/mass media. In the light of our results, it is important to highlight the importance of girl’s education for building awareness and empowerment for contraception through media, particularly social media.

## Data availability

Data used in this study are from the individual recode data file of the Paskitan 2019 Demographic and Health Survey, available from the Demographic and Health Survey (DHS) website. Access to the dataset requires registration and is granted only for legitimate research purposes. A guide for how to apply for dataset access is available at: https://dhsprogram.com/data/Access-Instructions.cfm.
