# Supplementary material for: Prevalence and determinants of contraception use in Pakistan: trend analysis from the Pakistan Demographic and Health Surveys (PDHS) dataset from 1990 to 2018
Source: F1000Res. 2021 Aug 11;10:790. [Version 1] doi: 10.12688/f1000research.55204.1 (PMC8411274; doi:10.12688/f1000research.55204.1)
Supplement: Peer review history

# Peer review: Reviewer response for version 1

**DOI:** 10.5256/f1000research.58761.r91662

**Published:** 2021-09-01

**Authors:** Nida Zahid

**Affiliations:** 1 Department of Surgery, Aga Khan University Hospital, Karachi, Pakistan

'In developing countries, pregnancy and childbirth are the leading causes of death among women. In this context, family planning and access to contraceptives are crucial for reducing pregnancy-related morbidity and mortality. The study aims to look into the trends of contraception and determinants of contraceptive use in Pakistan. The source of data is PDHS. The outcome variable is Contraception. The methodology of the study is robust and is described nicely by the authors. The statistical techniques applied by the authors are appropriate. The graphs nicely depict the results of the study

 Statistical analysis :

- Please mention the inferential tests that were used for assessing the association of categorical variables such as the chi-square/ Fisher exact test.
- Since the authors have mentioned that SPSS was used to analyze data, please remove the R software   

Results:

- Table1: Please recheck the p-value of work status.
- Please give a footnote and mention the significant variables and the test that was applied.
- It would be better to report the trend from 1990 to 2018  as a line graph instead of a table.
- Table 2: Please give footnotes indicating the significant variables and the test that was used

Is the work clearly and accurately presented and does it cite the current literature?

Yes

If applicable, is the statistical analysis and its interpretation appropriate?

Yes

Are all the source data underlying the results available to ensure full reproducibility?

Yes

Is the study design appropriate and is the work technically sound?

Yes

Are the conclusions drawn adequately supported by the results?

Yes

Are sufficient details of methods and analysis provided to allow replication by others?

Yes

Reviewer Expertise:

Mental Health, Public Health, Oncology, Male Infertility

I confirm that I have read this submission and believe that I have an appropriate level of expertise to confirm that it is of an acceptable scientific standard.

# Peer review: Reviewer response for version 1

**DOI:** 10.5256/f1000research.58761.r91664

**Published:** 2021-08-19

**Authors:** Adeel Khoja

**Affiliations:** 1 Department of Medicine, Aga Khan University Hospital, Karachi, Pakistan; 2 Adelaide Medical School, The University of Adelaide, South Australia, Australia

Overall, a very nicely written manuscript capturing and highlighting data on contraceptive use among the Pakistani population. I guess this study fills a good research gap in regard to LMICs.

 I personally feel, interviewing some women of reproductive age group from remote regions of Pakistan would have added some scientific rigour to this study and I foresee some sort of triangulation of results/findings from qualitative and quantitative study designs.

 Regarding the use of the PDHS dataset, was it obtained as a clean version, or do the authors have to undertake the cleaning and editing of the dataset, kindly mention this in the method section and also if relevant permissions were obtained for using the PDHS dataset from the relevant authorities.

 Were the seven categories of age mered into four categories based on the distribution of the data, kindly explain

 Please remove the last line from the method section regarding R software. It is not needed.

 I would encourage the authors to draw line graphs to show the trends of contraceptive use (which is increasing) with time (years) on x-ais.

 The wealth index was divided into five categories, can the authors please state the reason why it was divided into five, have the authors used any resource or literature backing to support these categories

Is the work clearly and accurately presented and does it cite the current literature?

Yes

If applicable, is the statistical analysis and its interpretation appropriate?

Yes

Are all the source data underlying the results available to ensure full reproducibility?

Yes

Is the study design appropriate and is the work technically sound?

Yes

Are the conclusions drawn adequately supported by the results?

Yes

Are sufficient details of methods and analysis provided to allow replication by others?

Yes

Reviewer Expertise:

Women and Children Health, mobile Health, electronic Health, e-Learning, Cardiology, Stroke, Non-Communicable Diseases

I confirm that I have read this submission and believe that I have an appropriate level of expertise to confirm that it is of an acceptable scientific standard.

# Response

**Published:** 2021-08-19

**Authors:** Zohra Lassi

**Affiliations:** University of Adelaide, Australia

Overall, a very nicely written manuscript capturing and highlighting data on contraceptive use among the Pakistani population. I guess this study fills a good research gap in regard to LMICs.

**Author's comment**: Thank you. 

 I personally feel, interviewing some women of reproductive age group from remote regions of Pakistan would have added some scientific rigour to this study and I foresee some sort of triangulation of results/findings from qualitative and quantitative study designs.

 Author's comment: This is a secondary analysis of the PDHS dataset and it involved no interaction or actual data collection in the field. 

 Regarding the use of the PDHS dataset, was it obtained as a clean version, or do the authors have to undertake the cleaning and editing of the dataset, kindly mention this in the method section and also if relevant permissions were obtained for using the PDHS dataset from the relevant authorities.

**Author's comment**: It has been mentioned in the data availability section at the very end. 

 Were the seven categories of age mered into four categories based on the distribution of the data, kindly explain

 Author's comment: It was merged into 4 categories because of small cell counts for some of the categories. 

 Please remove the last line from the method section regarding R software. It is not needed.

**Author's comment:** We were asked to mention this by the editor of the journal. 

 I would encourage the authors to draw line graphs to show the trends of contraceptive use (which is increasing) with time (years) on x-ais.

**Author's comment:** We do not think this is required as we have indicated and reported in the meta-analysis figure 3. 

 The wealth index was divided into five categories, can the authors please state the reason why it was divided into five, have the authors used any resource or literature backing to support these categories

**Author's comment**: This is how DHS generally categories wealth index and is uniform across all country that collects DHS data.
